# Study on the characteristics of genetic diversity of different populations of Guizhou endemic plant *Rhododendron pudingense* based on microsatellite markers

**DOI:** 10.1186/s12870-024-04759-5

**Published:** 2024-01-29

**Authors:** Shuang He, Congjun Yuan, Panli Zhang, Haodong Wang, Dali Luo, Xiaoyong Dai

**Affiliations:** 1 Guizhou Academy of Forestry, Guiyang, 550005 Guizhou China; 2 Guizhou Libo Karst Forest Ecosystem National Observation and Research Station, Libo, 558400 Guizhou China; 3 Guizhou Forestry School, Guiyang, 550200 Guizhou China; 4 Key Laboratory of National Forestry and Grassland Administration on Biodiversity Conservation in Karst Mountainous Areas of Southwestern China, Guiyang Guizhou, 55005 China

**Keywords:** *Rhododendron pudingense*, Genetic diversity, Populaitions structure, Microsatellite

## Abstract

**Background:**

*Rhododendron pudingense*, firstly discovered in Puding county of Guizhou province in 2020, have adapted to living in rocky fissure habitat, which has important ornamental and economic values. However, the genetic diversity and population structure of this species have been rarely described, which seriously affects the collection and protection of wild germplasm resources.

**Results:**

In the present study, 13 pairs of primers for polymorphic microsatellite were used to investigate the genetic diversity of 65 *R. pudingense* accessions from six different geographic populations. A total of 254 alleles (*Na*) were obtained with an average of 19.5 alleles per locus. The average values of polymorphic information content (*PIC*), observed heterozygosity (*Ho*), and expected heterozygosity (*He*) were 0.8826, 0.4501, and 0.8993, respectively, These results indicate that the microsatellite primers adopted demonstrate good polymorphism, and the *R. pudingense* exhibits a high level of genetic diversity at the species level. The average genetic differentiation coefficient (*Fst*) was 0.1325, suggested that moderate divergence occurred in *R. pudingense* populations. The average values of genetic differentiation coefficient and gene flow among populations were 0.1165 and 3.1281, respectively. The analysis of molecular variance (AMOVA) indicated that most of the population differences (88%) were attributed to within-population variation. The PCoA results are consistent with the findings of the UPGMA clustering analysis, supporting the conclusion that the six populations of *R. pudingense* can be clearly grouped into two separate clusters. Based on Mantel analysis, we speculate that the PD population may have migrated from WM-1 and WM-2. Therefore, it is advised to protect the natural habitat of *R. pudingense* in situ as much as possible, in order to maximize the preservation of its genetic diversity.

**Conclusions:**

This is the first comprehensive analysis of genetic diversity and population structure of *R. pudingense* in Guizhou province. The research results revealed the high genetic diversity and moderate population diferentiation in this horticulture plant. This study provide a theoretical basis for the conservation of wild resources of the *R. pudingense* and lay the foundation for the breeding or cultivation of this new species.

**Supplementary Information:**

The online version contains supplementary material available at 10.1186/s12870-024-04759-5.

## Background

*Rhododendron pudingense*, commonly called Puding Azalea, is an evergreen shrub belonging to the *Rhododendron* genus in the Ericaceae family. *R. pudingense* was first found in Puding county of Guizhou province in 2020, which possess a low number of individuals dispersedly distributed in small habitat areas [1].

Although previous studies have revealed that the Rhododendron species prefer to inhabit acidic soil, *R. pudingense* as a unique species in Guizhou can thrives in alkaline karst soils. *R. pudingense* plants were mainly distributed in four counties, such as Puding, Qinglong, Wangmo, and Zhenning [2]. Although *R. pudingense* have been discovered in multiple locations, they live in alkaline karst crevices, and seed reproduction rate of *R. pudingense* communities is low, with difficulties in the survival of seedlings. Based on the IUCN Red List criteria and standards (version 3.1), this species is defined as endangered (EN) and requires urgent research and conservation efforts [1]. Therefore, it is of utmost importance to protect this species, as both genetic resource conservation and plant breeding require assessment of the genetic diversity and outcomes of endangered species [3, 4]. To date, potential habitats, population structure, spatial distribution patterns, soil microbial community structure, functional diversity, and enzyme activities of *R. pudingense* plants have been reported in previous researchs [2, 5–7], the genetic diversity and population structure of *R. pudingense* population were still unclear, which may cause it difficult to plan conservation strategies for this *R. pudingense*.

Genetic diversity is an aspect of biodiversity that helps us understand conservation strategies for rare and endangered species [8]. For example, scholars have demonstrated through the summarization of molecular marker methods in studies on numerous species of angiosperms that endangered species possess lower genetic diversity compared to non-endangered species, and the loss of genetic diversity can cause loss of adaptive responses and evolutionary potential to complex environmental changes [9–11]. Studying the genetic diversity of endangered or rare and endemic species at the level of individuals or ecosystems can not only help understand the species’ evolutionary history and mechanisms for species endangerment, but also lay the foundation for developing scientifically effective protection measures. Furthermore, such studies can provide important guidance for the conservation of large-scale biodiversity and adaptation strategies for evolution. Therefore, quantifying genetic variation and diversity patterns within and between different populations is of paramount importance for the conservation and management planning of small population species.

Plant population genetics plays a crucial role in promoting plant breeding and conservation strategies. Several molecular marker methods, including microsatellite markers, also known as simple sequence repeats (SSR), Random Amplified Polymorphic DNA (RAPD), Amplified Fragment Length Polymorphism (AFLP), Inter Simple Sequence Repeat (ISSR), and Restriction Fragment Length Polymorphism (RFLP), were used to analyze genetic diversity in plants [12–15]. For genetic diversity evaluation, microsatellite markers have been widely applied in many plant species to evaluate genetic diversity, to construct genetic maps, and to determine species lineages due to four advantages: (1) high abundance, genome-wide coverage, and high polymorphism level; (2) high information content provided by multiple alleles at each locus; (3) Mendelian inheritance and co-dominance; (4) primers designed for each locus, facilitating collaboration and exchange between different laboratories. Microsatellite markers have been extensively used in genetic mapping, marker-assisted selection, variety identification, pedigree analysis, estimation of genetic distances among populations, and studies on evolution and genetic diversity [16]. In the present study, microsatellite markers were employed to analyze the genetic diversity indices, genetic differentiation, gene flow, and population structure of *R. pudingense* population collected from four different countries in Guizhou province. Our results aimed: (1) to systematically reveal genetic diversity and population structure of *R. pudingense* plants located in four countries in Guizhou province; (2) to explore the reasons of the current genetic patterns of this species; (3) to provide references for the conservation and breeding of germplasm resources of *R. pudingense*.

## Results

### Genetic charaterisitc of 13 microsatellite markers

In the present study, 13 primer pairs were selected for detection of genetic diversity of *R. pudingense* populations (Table 1). And a total of 254 alleles at 13 polymorphic microsatellite loci were amplified across 65 individual plants from 4 natural populations (Table 2). The number of alleles per locus ranged from 10 (RDW38) to 27 (RDW46 and RDE11) with an average of 19.5. The total number of effective alleles (*N*_*e*_*)* across all loci in this study was 139.6401, and the number of effective alleles per locus ranged from 4.1711 (RDW38) to 15.6260 (RDW46) with an average of 10.7415. The Shannon diversity index (*I*) ranged from 1.6605 (RDW38) to 2.9735 (RDW46) with an average of 2.5597. The polymorphic information content (*PIC*) ranged from 0.7241 (RDW38) to 0.9324 (RDW46), with an average of 0.8826, suggested that each locus exhibited high polymorphism (*PIC* ≥ 0.5). The observed heterozygosity (*H*_*o*_) ranged from 0.2787 (RDW31) to 0.6667 (R557) with an average of 0.4501. The expected heterozygosity (*H*_*e*_) ranged from 0.7662 (RDW38) to 0.9435 (RDW46) with an average of 0.8993.

In addition, the within-population inbreeding coefficient (*F*_*is*_) ranged from 0.1784 (R557) to 0.6036 (RDW31), with an average of 0.4169. The total inbreeding coefficient (*F*_*it*_) ranged from 0.2424 (R557) to 0.6661 (RDW31), with an average of 0.4942. The genetic differentiation coefficient (*F*_*st*_) ranged from 0.0779 (R557) to 0.2150 (N8), with an average of 0.1325, indicating that only 13.25% of the genetic variation occurred between populations, while the remaining 86.75% occurred within populations, suggesting the presence of inbreeding among the selected genetic loci. The gene flow (*N*_*m*_) ranged from 0.9127 (N8) to 2.9589 (R557), with an average of 1.6366. Among them, N8 (*N*_*m*_=0.9127) was less than 1, indicating that the population at this locus is more prone to genetic drift and differentiation (Table 2).

**Table 1:** Information of 13 pairs of polymorphic microsatellite primers

Locus	forward sequence(5’—3’)	Repetit motif	Fluorescence	Size/bp
RDW1	GCCTCTAACTACTTGCTCCA	(TC)9	HEX	200–290
RDW16	GGTGATCGTGTCGGAATACA	(GA)9	HEX	270–300
RDW31	AAGGTGATCGTGTCGGAATA	(GA)8	FAM	260–290
RDW35	TAAGGTTGGTGTAGCGTGTA	(TC)5(CT)6(ATA)3	FAM	260–300
RDW38	GTGTTTGAAATTGTCGGC	(TAGAG)4(AG)7(AGAGAT)3	ROX	110–140
RDW46	TCTCCAGAAGTACGCAAAT	(CTT)3(GA)11	HEX	310–370
R140	GAAGCCAGTGCTGTGATT	(AG)6	TAMRA	110–160
R299	TACTGTGCTTAGACGCCATT	(AG)12	TAMRA	90–130
R557	CGAAACTCAGAACCTCCG	(CT)9(TG)6	ROX	190–230
N8	CGGAGAGTGATGAAACAGAA	(AG)19(TG)8	TAMRA	90–130
N73	GCAACCTACATTCTCAACAT	(AC)3 C(CA)6	FAM	180–220
RD8	AACCTCCTCAAATCGACAAC	(CT)14	FAM	110–170
RDE11	TAATCCAGACTATCCAGTGC	(CT)7	ROX	140–270

**Table 2:** Genetic charateristics of 13 pairs of microsatellite markers

Locus	N_a_	N_e_	I	PIC	H_o_	H_e_	F_is_	F_it_	F_st_	N_m_
RDW1	16	7.7039	2.3690	0.8595	0.2951	0.8774	0.6035	0.6620	0.1476	1.4443
RDW16	15	7.1854	2.2512	0.8478	0.2923	0.8675	0.5975	0.6575	0.1492	1.4253
RDW31	15	6.1100	2.1846	0.8219	0.2787	0.8432	0.6036	0.6661	0.1576	1.3361
RDW35	19	13.4984	2.7460	0.9211	0.5385	0.9331	0.3160	0.4215	0.1542	1.3714
RDW38	10	4.1711	1.6605	0.7241	0.5156	0.7662	0.2641	0.3247	0.0823	2.7865
RDW46	27	15.6260	2.9735	0.9324	0.6508	0.9435	0.1912	0.3048	0.1404	1.5303
R140	26	11.2817	2.7781	0.9055	0.3846	0.9184	0.5394	0.5768	0.0813	2.8239
R299	21	12.1583	2.7308	0.9123	0.5077	0.9249	0.3825	0.4603	0.1259	1.7358
R557	22	13.6800	2.8212	0.9223	0.6667	0.9351	0.1784	0.2424	0.0779	2.9589
N8	15	8.3384	2.3226	0.8687	0.2903	0.8872	0.5691	0.6618	0.2150	0.9127
N73	20	13.0000	2.7285	0.9180	0.6308	0.9302	0.2367	0.3210	0.1105	2.0130
RD8	21	15.0356	2.8391	0.9296	0.3692	0.9407	0.5673	0.6087	0.0956	2.3643
RDE11	27	11.8513	2.8706	0.9106	0.4308	0.9227	0.4306	0.5357	0.1847	1.1038
Mean	19.5	10.7415	2.5597	0.8826	0.4501	0.8993	0.4169	0.4942	0.1325	1.6366

*N*_*a*_: The total number of observed alleles per locus; *N*_*e*_: The efective number of alleles; *I*: Shannon’ information index; *PIC*: Polymorphism information content; *H*_*o*_: Observed heterozygosity; *H*_*e*_: Expected heterozygosity; *F*_*is*_: Inbreeding coefficient within population; *F*_*it*_: Total inbreeding coefficient; *F*_*st*_: Genetic differentiation coefficient; *N*_*m*_: gene flow

### Genetic diversity of populations in *R. pudingense*

To further investigate the genetic diversity among different population, we performed comparative analysis the genetic diversity of six populations based on microsatellite markers (Table 3). Our results showed that the number of allele loci in the six populations ranged from 5.9231 (PD) to 8.2308 (WM-3) with an average of 7.5. The range of effective alleles (*N*_*e*_) ranged from 3.8988 (PD) to 6.0061 (WM-3) with an average of 5.1477. This result indicated differences in genetic diversity among different populations. The values of the Shannon diversity index (*I*) range from 1.4842 (PD) to 1.8751 (WM-3), with an average of 1.7414, and the differences between the populations are small, indicating relatively similar levels of genetic diversity among the populations. The observed heterozygosity (*H*_*o*_) ranges from 0.3706 (WM-2) to 0.5058 (ZN), with an average of 0.4515. The expected heterozygosity (*H*_*e*_) ranges from 0.7188 (PD) to 0.8073 (WM-3), with an average of 0.7808, this suggests that the genetic diversity of different populations *R. pudingense* in this study is relatively rich. Furthermore, *H*_*o*_ is lower than *He* in all populations, indicating a certain degree of heterozygote deficiency within the populations. Combining the expected heterozygosity and Shannon diversity index, WM-3 population has the highest genetic diversity, while PD has the lowest genetic diversity. The values of fixation index (*F*) range from 0.3622 (QL) to 0.5466 (WM-2), all greater than 0.25, and with an average of 0.4258, indicating the presence of inbreeding within different populations of *R. pudingense*, and the and the inbreeding phenomenon in the WM-2 population is more severe, and more prone to genetic differentiation compared to other populations. In addition, we also tested whether 13 pairs of microsatellite loci followed the Hardy-Weinberg equilibrium in the central distribution of the *R. pudingense*g. The results showed that most loci deviated from genetic equilibrium, indicating that *R. pudingense* populations was not in Hardy-Weinberg equilibrium. In the linkage disequilibrium test, no significant linkage disequilibrium was found between any pair of loci. Therefore, the reason why the natural population of *R. pudingense* does not follow the random mating pattern may be due to the existence of null alleles at loci, leading to insufficient heterozygote individuals (Table 4).

**Table 3:** Genetic diversity of 6 populations of *R. pudingense*

Populaition	N_a_	N_e_	I	H_o_	H_e_	F
ZN	8.0000	5.3739	1.7973	0.5058	0.7800	0.3726
PD	5.9231	3.8988	1.4842	0.4406	0.7188	0.3946
QL	7.3846	4.6123	1.6732	0.4892	0.7557	0.3622
WM-1	7.9231	5.2907	1.7955	0.4338	0.7815	0.4541
WM-2	7.7692	5.7045	1.8230	0.3706	0.8045	0.5466
WM-3	8.2308	6.0061	1.8751	0.4692	0.8073	0.4248
Mean	7.5	5.1477	1.7414	0.4515	0.7746	0.4258

*N*_*a*_: The total number of observed alleles per locus; *N*_*e*_: The effective number of alleles; *I*: Shannon’ information index; *H*_*o*_: Observed heterozygosity; *H*_*e*_: Expected heterozygosity; *F*: Fixed index; ZN: Zhening; PD: Puding; QL: Qinglong; WM: Wangmo

**Table 4:** Hardy-Weinberg equilibrium test of 6 populations of *R. pudingense*

Population	HWE-P
ZN	RDW1***, RDW16**, RDW31**, RDW38**, R299***, N8***, N73**, RD8***, RDE11***
PD	RDW1**, RDW16***, RDW31***, RDW35***, R557**, N8**, N73*, RD8***, RDE11**
QL	RDW1***, RDW16***, RDW31***, RDW35*, RDW38**, R140***, R299**, R557***, N8**, RD8**, RDE11**
WM-1	RDW1****, RDW16***, RDW31***, RDW35***, RDW46**, R140***, R299***, N8**, N73***, RD8***, RDE11**
WM-2	RDW1***, RDW16***, RDW31***, RDW35***, RDW38**, R140***, R299**, N8***, N73***, RD8***, RDE11***
WM-3	RDW1***, RDW16**, RDW31***, RDW35*, RDW38*, RDW46*, R140***, R299***, R557**, N8***, RD8***

*: *P* < 0.05. **: *P* < 0.01. ***: *P* < 0.001 (loci with heterozygote deficit)

### Genetic differentiation and gene flow among populations in *R. pudingense*

Analysis of molecular variance showed that that 88% of the genetic variation mainly come from between-individual variations within the population variation, while only 12% is due to between-population variation (Table 5).

And the values of the genetic differentiation between different populations ranges from 0.0125 (between WM-1 and WM-2) to 0.1830 (between QL and ZN), with an average of 0.1165 (Table 6), which is consistent with the result of AMOVA, indicating that the genetic variation in *R. pudingense* populations is mainly caused by between-individual variation within the populations. Moreover, the gene flow associated with genetic differentiation ranges from 1.1162 (between ZN and QL) to 19.7687 (between WM-1 and WM-2) among different populations, with an average of 3.1281 (Table 6), indicating that there is significant gene flow between *R. pudingense* populations. This results further revealed that genetic differentiation among populations is inhibited. This is also a reason for the relatively low genetic differentiation in the populations of *R. pudingense*.

To further evaluate the genetic divergence between populations, Popgen32 software was used to calculate the genetic distance between *R. pudingense* populations (Table 7). The genetic distance between six *R. pudingense* populations ranges from 0.4315 (between WM-1 and WM-2) to 1.6190 (between QL and ZN), with an average of 1.0410, and the coefficient of genetic similarity ranges from 0.1981 (between QL and ZN) to 0.6495 (between WM-1 and WM-2), with an average of 0.3714, indicating that the farthest genetic relationships with QL and ZN, while between WM-1 and WM-2 are the closest.

**Table 5:** Analysis of molecular variance (AMOVA) for 6 populations of *R. pudingense*

Source	df	Sum of Squares	MS	Est. Var.	Ratio of variance(%)
Among Populations	5	196.169	39.234	2.143	12
Within Populations	59	947.769	16.064	16.064	88
Total	64	1143.938		18.207	100

**Table 6:** Result of gene flow *N*_*m*_ (upper triangle) and genetic differentiation results coeffcient (lower triangle) between populations. Bold character indicates the highest value, while italic bold character displays the lowest value

	ZN	PD	QL	WM-1	WM-2	WM-3
**ZN**	-	1.3618	***1.1162***	1.5495	2.0536	1.7886
**PD**	0.1551	-	1.3305	2.1400	2.0681	1.3359
**QL**	**0.1830**	0.1582	-	2.7664	2.0995	1.4448
**WM-1**	0.1389	0.1046	0.0829	-	**19.7687**	2.1808
**WM-2**	0.1085	0.1078	0.1064	***0.0125***	-	3.9173
**WM-3**	0.1226	0.1576	0.1475	0.1028	0.0600	-

**Table 7:** Result of genetic identity between populations (upper triangle) and genetic distance (lower triangle) between populations. Bold character indicates the highest value, while italic bold character displays the lowest value

	ZN	PD	QL	WM-1	WM-2	WM-3
**ZN**	-	0.3536	***0.1981***	0.2203	0.2922	0.2860
**PD**	1.0396	-	0.3957	0.4968	0.4463	0.2859
**QL**	**1.6190**	0.9270	-	0.5141	0.4008	0.2687
**WM-1**	1.5128	0.6995	0.6653	-	**0.6495**	0.3206
**WM-2**	1.2302	0.8067	0.9142	***0.4315***	-	0.4428
**WM-3**	1.2517	1.2523	1.3143	1.1375	0.8147	-

### Population structure and genetic relationships

**Fig. 1:**
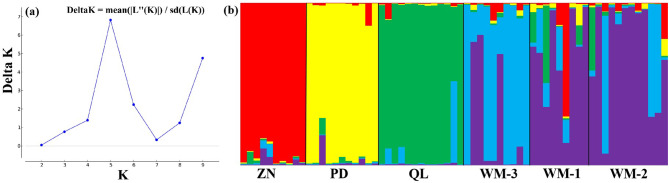
(**a**) The ΔK method of STRUCTURE analysis plots the change of K values; (**b**) STRUCTURE analysis of *R. pudingense* in Guizhou province of China based on 13 microsatellite markers

**Fig. 2:**
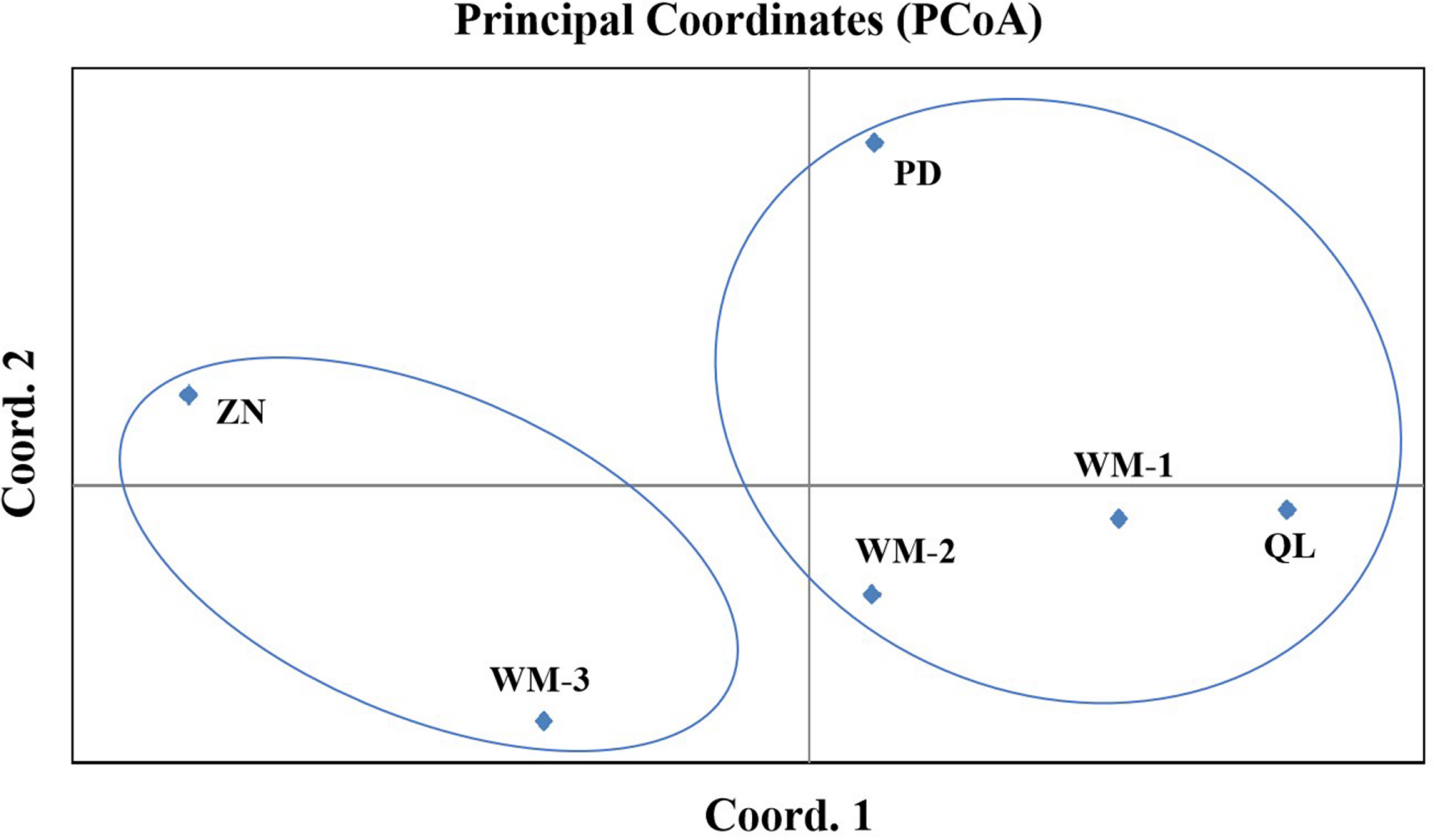
Principal coordinate analysis (PCoA) for 6 populations of *R. pudingense*

The result revealed that maximum value of delta K was at K = 5, thus, the all six populations in this study can be divided into five genetic clusters (Fig. 1; Table S1). The genetic composition of Zhenning (ZN) contained 10 individuals, mainly originates from genetic cluster 5 (red squares). The genetic composition of Puding (PD) contained 11 individuals, mainly originates from genetic cluster 4 (yellow squares).The genetic composition of Qinglong (QL) contained 13 individuals, mainly originates from genetic cluster 3 (green squares), and the genetic composition of Wangmo (WM) contained 31 individuals, among which the genetic composition of WM-3 includes 10 individuals, mainly from originates cluster 2 (blue square), and the genetic compositions of WM-1 and WM-2 include 10 and 11 individuals respectively, mainly from originates cluster 1 (purple square)(Fig. 1b). Although most individuals are assigned to different genetic clusters, it should be noted that the populations are not completely independent from each other. To further assess the genetic relationships among six *R. pudingense* populations, principal coordinate analysis (PCoA) was performed based on Nei’s genetic distance for the 6 populations (Figs. 2) and 65 plant samples (Fig. S1). The cumulative variance percentage of the first three axes was 17.86% (Axis 1–7.88%, Axis 2–5.12%, Axis 3–4.86%) (Fig. 2). The closer the distance between two populations in the graph, the smaller genetic background differences between them. And the results of the PCoA were consistent with those of the structure analysis and supported the UPGMA clustered tree, as described below.

**Fig. 3:**
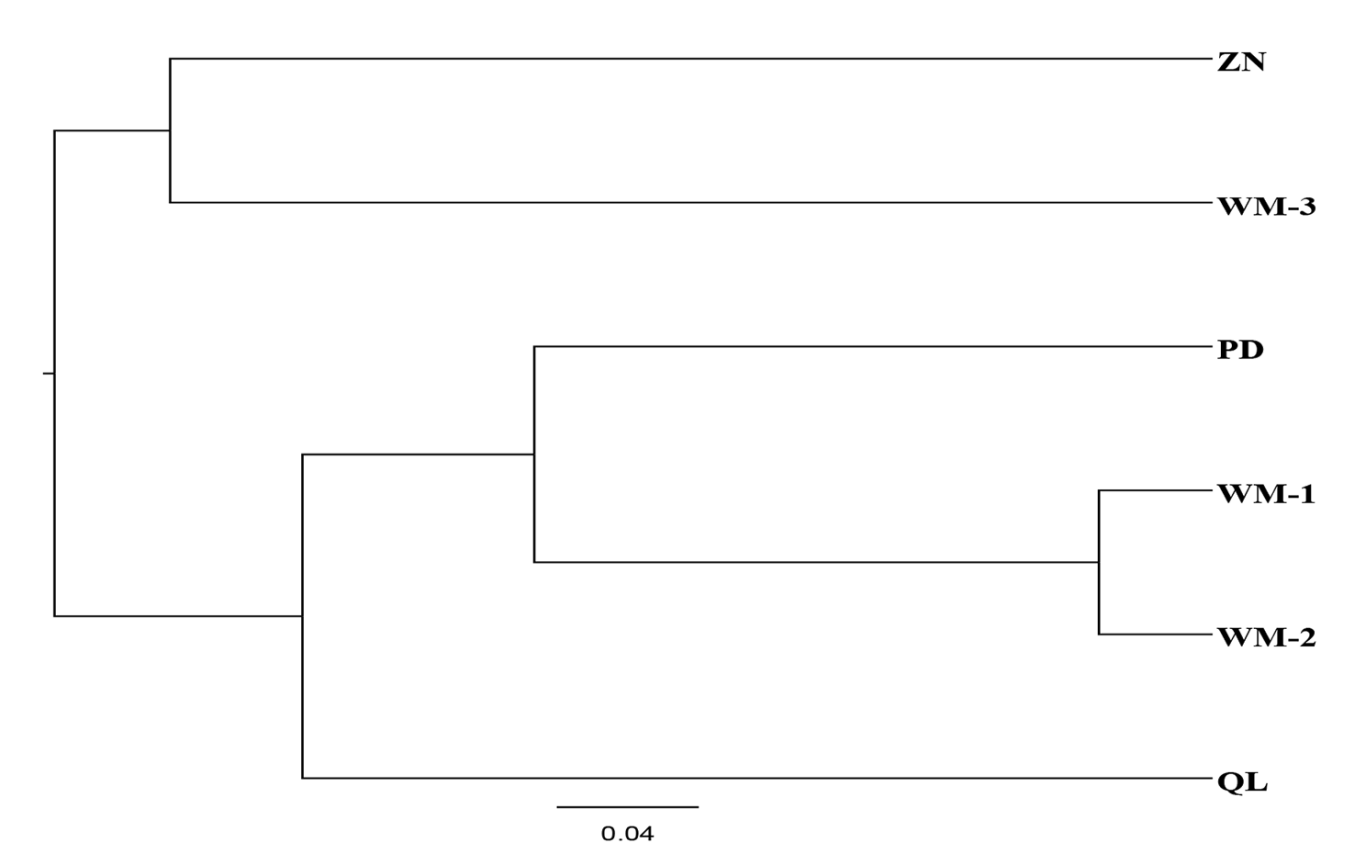
UPGMA clustering results for six populations

The UPGMA dendrogram was constructed from Nei’s genetic distance values and is an accurate reflection of the genetic relationships among and within populations (Fig. 3). The UPGMA dendrogram indicated that the six *R. pudingense* populations could be divided into two major clusters (Fig. 3). This clustering result is consistent with the results of genetic similarity and genetic distance between populations.

**Fig. 4:**
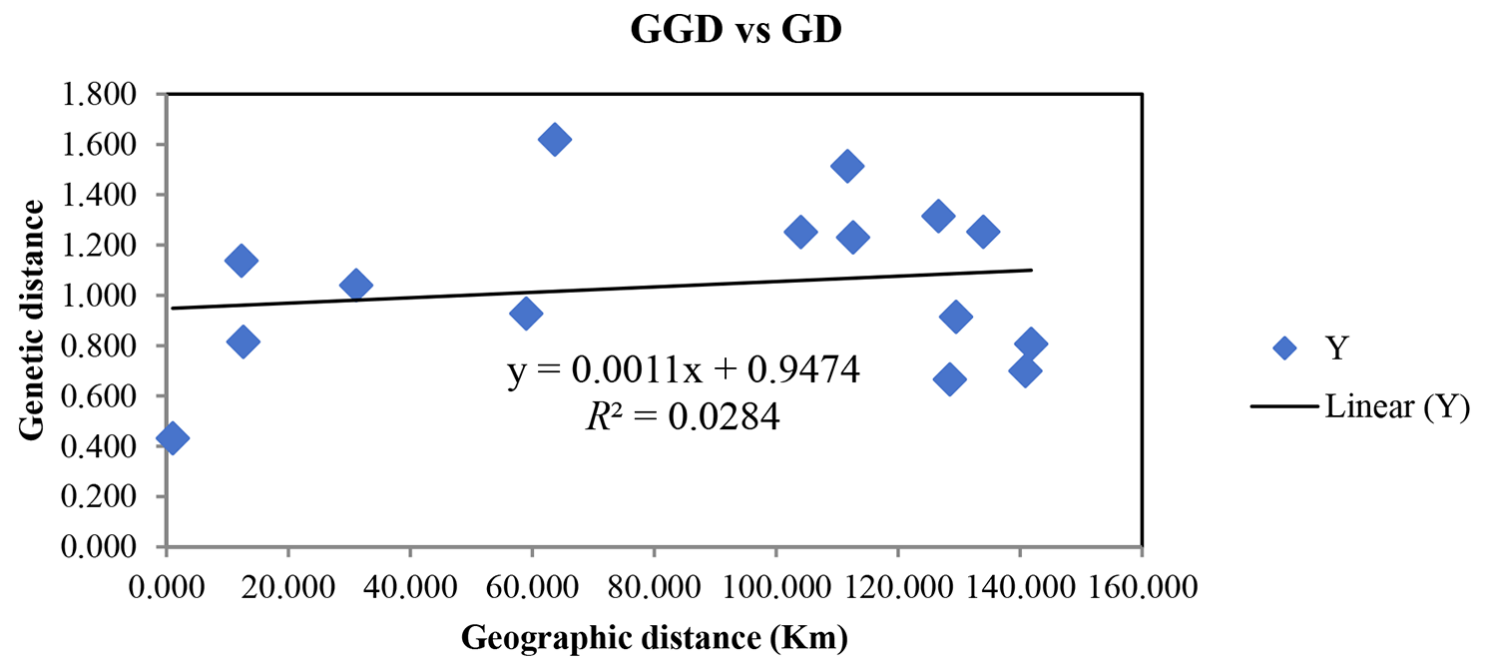
Correlation test of genetic distance (GD) and geographic distance (GGD) for 6 populations of *R. pudingense*

A Mantel test conducted for *R. pudingense* indicated correlation between genetic distance and geographic distance among populations was not significant (*r* = 0.1685, *P* = 0.210) (Fig. 4), this suggests that genetic differentiation among *R. pudingense* was not caused by geographic distance .

## Discussion

The magnitude of genetic diversity is a product of long-term biological evolution and serves as a prerequisite for the survival, development, and evolution of organisms. Maintaining genetic diversity within natural populations is crucial for ensuring the continued survival, fitness, and evolutionary potential of a species [10]. Traditionally, diversity has been evaluated through analysis of morphological and physiological traits in plants. However, due to the instability of these traits under different environmental conditions, and as the size of plant populations decreases, loss of genetic diversity reduces their ability to adapt to changes in the environment, with inbreeding and reduced fitness inevitable consequences for most species.In recent years, the development of molecular DNA molecular marker methods has advanced our understanding of genetic resources [17–21]. And among a rang of DNA molecular marker techniques, microsatellite molecular marker technology is widely regarded as the most practical method in population genetic studies due to its ability to measure codominant alleles and exhibit a high level of polymorphism [22]. Several studies have been conducted on the genetic diversity of the *Rhododendron* genus using microsatellite technology [23–26]. This study is the first to investigate the genetic diversity and population structure of *R. pudingense* through molecular marker technology, it is important for the protection, management and understanding of their genetic relationships.

### Genetic diversity of populations in *R. pudingense*

Differences in genetic diversity are influenced by several factors, such as unique evolutionary history, distribution patterns, and human-induced disturbances. Higher genetic diversity or greater genetic variation indicates a stronger adaptive capacity of organisms to environmental changes. Understanding the magnitude, spatial and temporal distribution, and relationship with environmental conditions of within-species variation helps us further conserve rare or endangered species [27].

In this study, microsatellite marker was used to amplify 65 samples from the four populations of *R. pudingense* using 13 primer pairs. A total of 254 allele loci were amplified, and the *F*_*is*_ and *F*_*it*_ values for the selected primers were both greater than 0, indicating a low level of hybridization among the 13 microsatellite loci [28, 29], and that indicating high genetic diversity within the population. Additionally, the average Shannon’s information index (*I*) for the 13 primers was 2.5597, and the average expected heterozygosity (*H*_*e*_) was 0.8993, the average eobserved heterozygosity (*H*_*o*_) was 0.4501. In general, a higher genetic diversity index indicates a greater diversification of the genome within a population and a greater amount of genetic variation among individuals [10]. The results of this study are higher than the findings of [22] in terms of genetic diversity using microsatellite markers (*H*_*o*_=0.450, *H*_*e*_=0.899, *I* = 2.560). These results indicate the high polymorphism of the microsatellite marker technology and support the feasibility of using microsatellite markers to investigate the genetic diversity of *R. pudingense*.

Indices such as polymorphic information content (*I)*, observed heterozygosity (*H*_*o*_) [30], expected heterozygosity (*H*_*e*_) [31], and fixation index (*F*) [32] are commonly used to describe population genetic diversity. The expected heterozygosity (*H*_*e*_) values for the populations of *R. pudingense* ranged from 0.7188 to 0.8073, and the observed heterozygosity (*H*_*o*_) values ranged from 0.3706 to 0.5058. In all six populations, *H*_*e*_ was greater than *H*_*o*_, and *F* were greater than 0, this indicates the presence of some degree of inbreeding in the population of *R. pudingense*. And these results are consistent with previous studies on the genetic structure of endemic plants by using microsatellite technology [24, 25, 30]. Based on these findings, we speculate that there is a high level of inbreeding within the populations of *R. pudingense*, which increases population homozygosity and exacerbates genetic differentiation among populations. According to the principles of conservation genetics, inbreeding can reduce the survival and reproductive capacity of a species, and leading to low genetic diversity, that may cause a population to decline [10, 33]. Therefore, when conserving *R. pudingense*, the phenomenon of population inbreeding should also be taken into consideration.

Generally, a small population size, narrow distribution range, large spatial distance and high altitude between populations can restrict pollination between groups, leading to self-fertilization or inbreeding and even reducing genetic diversity [34, 35]. In this study, the PD population is situated at an altitude of approximately 1500 m, where they face the challenges of a harsh mountain environment. This high-altitude region imposes significant selection pressure on the PD population. Moreover, the lack of birds and animals that aid in seed dispersal makes it difficult for them to engage in gene exchange with the outside world [34]. Additionally, the high humidity in the mountain air hinders pollen from being carried long distances by the wind, further limiting internal genetic exchange within the population. Consequently, these factors contribute to a reduced level of genetic diversity within the PD population. The result also suggest a diminished capacity of the population to adapt to evolving environments, consequently elevating the risk of species extinction [10, 19].

### Genetic differentiation and gene flow among populations in *R. pudingense*

The genetic differentiation coefficient (*F*_*st*_) and gene flow (*N*_*m*_) are commonly used indices to describe the degree of differentiation between natural populations [36, 37]. If gene flow is low, there is limited genetic exchange between the two populations, resulting in a high genetic differentiation coefficient and a distant genetic relationship between the populations [38, 39] and [33] suggested that when the *F*_*st*_ coefficient ranges from 0.05 to 0.15, natural populations are at a moderate level of differentiation. In this study, the average genetic differentiation coefficient (*F*_*st*_) of *R. pudingense* was 0.1138, indicating a low degree of genetic differentiation between the populations. Gene flow (*N*_*m*_) refers to the transfer and exchange of genes between different populations, and it can weaken the genetic differences between populations [40]. Generally, when *N*_*m*_ > 1, gene flow is high, and the degree of genetic differentiation between populations is low, allowing populations to resist the effects of genetic drift. When *N*_*m*_ < 1, populations with smaller numbers are more likely to undergo genetic drift [41]. In this study, the average *N*_*m*_ of *R. pudingense* populations was 3.1281, and the gene flow between the WM population and the other three populations was relatively high, with values exceeding 2, theoretically preventing genetic differentiation caused by genetic drift [27]. This result suggests frequent gene flow between the WM-1,WM-2,WM-3 populations and the other three populations. However, genetic drift is not the primary factor influencing changes in plant genetic outcomes, and populations are also affected by habitat fragmentation and destruction. Genetic drift may gradually occur as a result [42]. Meanwhile, the results of molecular variance analysis in this study indicated that 88% of the genetic variation originated within populations, while 12% of the genetic variation originated among populations (Table 5), which is consistent with the results of gene flow and genetic differentiation. The AMOVA results also support population differentiation. AMOVA reveals molecular differences between populations and within populations, primarily highlighting molecular differences within populations rather than between populations. This situation is the same as in the studies of [24] and [25] using microsatellite markers. The magnitude of genetic variation is influenced by multiple factors, and complex ecological environments are one of the reasons for genetic variation in species. Among them, the Ericaceae is an outcrossing plant, and its floral scent can attract insects for pollination, facilitating gene flow between different populations [34].

### Population structure and genetic relationships

In genetic diversity analysis, multiple methods are usually used in combination to obtain a more comprehensive result and interpretation [21, 43–45]. The PCoA and UPGMA analyses have the ability to cluster populations based on genetic distance or dissimilarity, constructing a hierarchical clustering dendrogram that visually represents the differences between samples, and the STRUCTURE software can infer population genetic structure using genetic markers. In this study, the UPGMA clustering analysis divided six populations from four different regions into two distinct clusters, indicating the presence of two separate genetic populations in these areas. and the results of the PCoA analysis are in agreement with the STRUCTURE plot and support the UPGMA clustering tree, further validating these findings.

Furthermore, the UPGMA clustering analysis revealed that PD is genetically closer to WM-1 and WM-2, indicating a close genetic affinity despite the geographical distance between these populations. The Mantel analysis results highlighted a non-significant correlation between geographical distance and genetic distance (*r* = 0.1685, *P* = 0.210). Based on the findings of gene flow, genetic differentiation, and the Mantel test analysis, it can be hypothesized that the PD population migrated from WM-1 and WM-2.

### Conservation of populations

The genetic diversity of organisms forms the basis for their adaptation to dynamic environments. The higher the genetic diversity or the greater the genetic variation within a species, the stronger its capacity to adapt to environmental changes, expand its distribution range, and explore new habitats. It is evident that the evolutionary potential, resistance to adverse environments, ecosystem resilience, and stability of a species are all contingent upon the magnitude of genetic diversity [26, 46, 47]. Although *R. pudingense* exhibits high genetic diversity, its distribution range is actually narrow, and the population is small. There is high genetic variation within populations of *R. pudingense*, but low genetic differentiation among populations, and the correlation between genetic distance and geographic distance is not significant. Therefore, the following suggestions are proposed: First, it is recommended to expand the habitat of *R. pudingense* and conduct large-scale regional protection efforts. Second, suitable locations can be selected based on the habitat requirements of *R. pudingense* for seedling cultivation. Third, under favorable conditions, efforts can be made to domesticate and cultivate *R. pudingense*, expanding its ecological niche and enabling it to survive and reproduce in a more diverse environment.

## Conclusions

The genetic information from this study offers primary data for understanding the genetic diversity and population structure of *R. pudingense*, which can contribute to the formulation of conservation and management measures for endangered plants. Natural populations showed moderate to high levels of genetic diversity, high gene flow, and low genetic differentiation among populations. These populations serve as valuable genetic resources for future breeding programs and conservation strategies. This is the first study to utilize microsatellite markers to investigate the genetic diversity of *R. pudingense*, providing valuable references for improving germplasm resources and parental selection in breeding strategies.

The markers used in this study can be used to study population structure, genetic diversity, germplasm resource collection, and conservation strategies. They provide important information on genetic structure and contribute significantly to future improvements, provides a deeper understanding of the reasons for the endangerment of *R. pudingense* and offers scientific support for the conservation of its genetic resources.

## Methods

### Plant materials

**Fig. 5:**
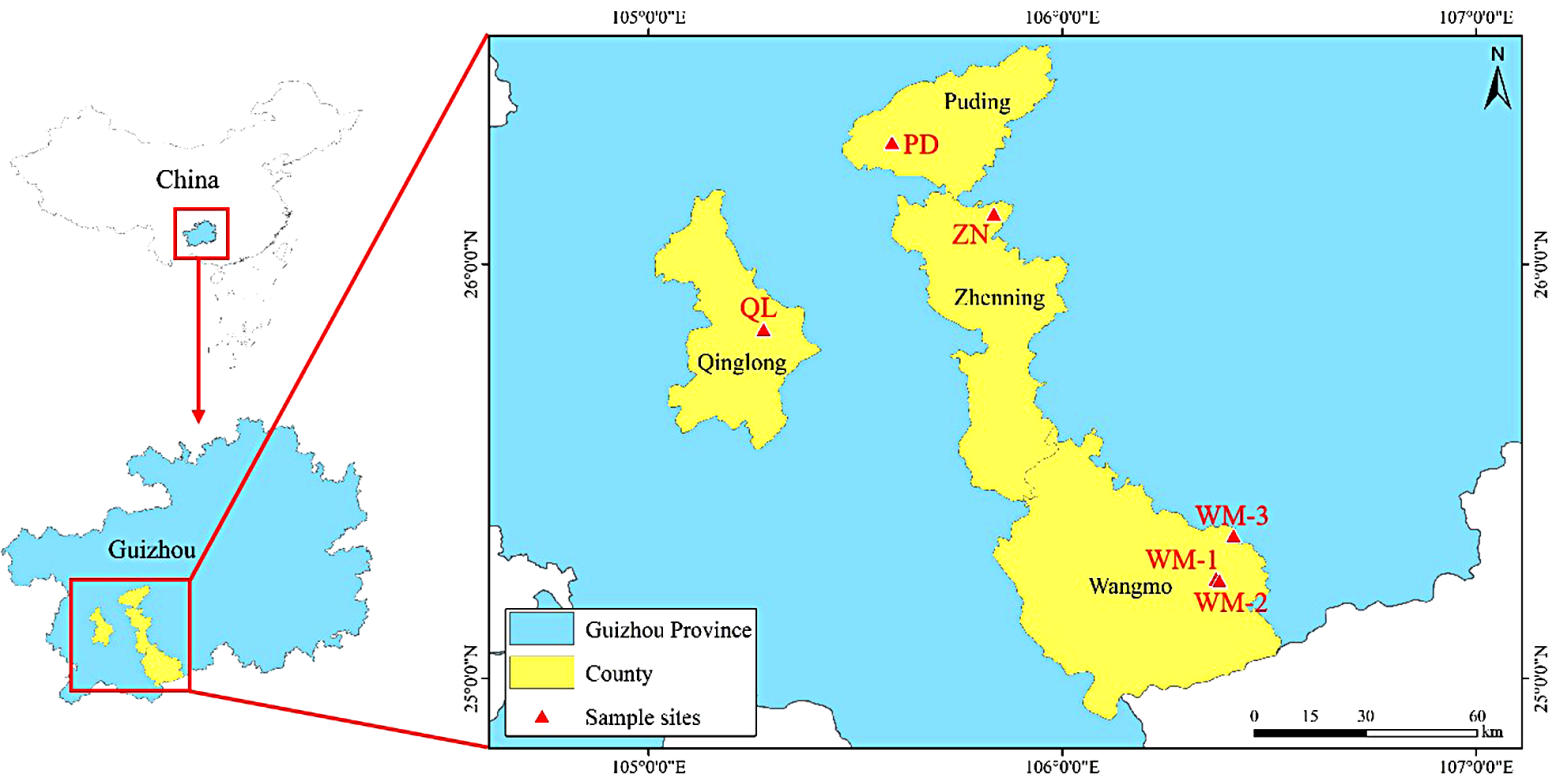
Geographic locations of *R. pudingense* populations sampled in this study

The 65 *R. pudingense* materials used in this study were collected from six different populations in Guizhou province. Specifically, 11 samples were collected from PD county, 13 samples from QL county, 31 samples from WM county, and 10 samples from ZN county. Details of the sampling are listed in Table 8; Fig. 5. Fresh leaves were collected and then labeled with sample numbers, then placed in sealed preservation bags and stored in a cooler box for transportation back to the laboratory. The samples were subsequently frozen rapidly using liquid nitrogen and stored at -80 degrees Celsius in a freezer for DNA extraction. The formal identification of the samples used in this study was performed by Xiao-Yong Dai. Voucher specimens were deposited in the Herbarium of Guizhou Provincial Academy of Forestry (GF) and Kunming Institute of Botany, Chinese Academy of Sciences (PE), the deposition number was 180,507,112 (fl., Holotype GF!, isotypes KUN!, PE!). Our field investigation and experimental studies comply with the regulations of local legislative bodies, national and international guidelines.

**Table 8:** The sampling information of 6 populations of *R. pudingense*

Population	Latitude(°N)	Longitude(°E)	Altitude(m)	Sample size
ZN	26.12094535°N	105.83423407°E	1394	10
PD	26.29305958°N	105.58832058°E	1504	11
QL	25.84168538°N	105.27780832°E	1421	13
WM-1	25.24084252°N	106.37148642°E	1303	10
WM-2	25.23547270°N	106.37980705°E	1310	11
WM-3	25.34461480°N	106.41384152°E	1221	10

### DNA extraction and PCR amplification

The plant genomic DNA was extracted from leaf samples using the plant Genomic DNA extraction Kit (200) (Qingke Biotech Co., Ltd.; TSP102-200). a total of 13 pairs of highly polymorphic and amplified microsatellite primers [48–51] were screened by combining with the *R. pudingense* materials, which served as amplification primers for subsequent analysis. Based on the 13 pairs of polymorphic primers, forward primers were synthesized with FAM, HEX, ROX, and TAMRA fluorescent labels at the 5’ end, and all 65 samples were amplified and analyzed by capillary electrophoresis. The amplification system was as follows: The PCR reaction system consists of 17 µl of Golden Mix (Green), 1 µl of 10 µM Primer F, 1 µl of 10 µM Primer R, and 1 µl of Template (gDNA), making a total volume of 20 µl. The PCR reaction program is as follows: 98 °C for 2 min, one cycle; 98 °C for 10 s, annealing temperature (Tm) for 10 s, 72 °C for 10 s, 35 cycles; 72 °C for 5 min, one cycle. The amplified PCR products are subjected to agarose gel electrophoresis (2 µl of the sample + 6 µl of bromophenol blue) at 300 V for 12 min to obtain the gel image. The gel image is used to determine the template concentration, and then it is diluted with water to the required concentration for capillary electrophoresis. Finally, specific and polymorphic loci with high specificity and good polymorphism are selected for statistical analysis.

### Data analyses

The specific bands of each individual were counted based on their band sizes (bp). The peaks were analyzed using GeneMapper 4.1 software [52], with signal values above 400 and no other interfering peaks. Additionally, the peaks obtained from the same locus exhibited similar shapes. If the peak shapes were dissimilar, even if the peak value exceeded 400 and there was no other interfering peak, the data were not considered acceptable. This criterion was used to filter out usable data and establish the raw data matrix.

The population genetic structure was analyzed using the Bayesian model-based clustering method in STRUCTURE version 2.3.3 software [53]. The Markov Chain Monte Carlo (MCMC) method was employed, allowing for pre-defined population grouping (K) and calculating, sampling, and assigning individuals based on allele frequencies. The parameter settings were as follows: K values ranged from 1 to 10, with 10 independent runs for each K value, and a total of 100,000 iterations per run for repeated sampling. Finally, the most suitable K value was determined based on the method described in [54] using STRUCTURE HARVESTER (http://taylor0.biology.ucla.edu/struct_harvest/) website. The Hardy-Weinberg equilibrium (HWE) of each population at each locus was evaluated using the online software Genepop v.4.7 (https://genepop.curtin.edu.au/) [55].

Based on this criterion, genetic distances were calculated using the unweighted pair-group method with arithmetic means (UPGMA) to construct a clustering tree of individuals [56]. Specifically, the UPGMA tree was built using the populations-1.2.30 software, with the value set to 1000. The visualization and editing of the clustering tree were carried out using FigTree version 1.4.2 software.

According to the results of the analysis of population genetic structure, variation and differentiation between populations were calculated using GenAlEx version 6.5 software. Significance tests were conducted [56]. Gene flow (*N*_*m*_) was calculated using [41] formula: *N*_*m*_ = 0.25(1 - *F*_*st*_)/*F*_*st*_.

To further investigate the genetic relationship among individuals of *R. pudingense* in four different populations, principal coordinate analysis (PCoA) was conducted using GenAlEx v6.5 [57].

### Electronic supplementary material

Below is the link to the electronic supplementary material.

Supplementary Material 1

## Data Availability

The sequencing data of the 13 polymorphic microsatellite primers were listed in the manuscript, and no other DNA sequences were applied to this study.

## Abbreviations

AFLP: Amplified fragment length polymorphism
AMOVA: Analysis of molecular variance
ISSR: Inter simple sequence repeat
PCoA: Principal coordinate analysis
RAPD: Random amplified polymorphic DNA
RFLP: Restriction fragment length polymorphism
ISSR: Inter simple sequence repeat
SSR: Simple sequence repeat
