# Supplementary material for: Study on the characteristics of genetic diversity of different populations of Guizhou endemic plant Rhododendron pudingense based on microsatellite markers
Source: BMC Plant Biol. 2024 Jan 29;24:77. doi: 10.1186/s12870-024-04759-5 (PMC10823706; doi:10.1186/s12870-024-04759-5)

**Table S1** Sample proportions of each population at K=5 in STRUCTURE

| K=5 | 1 | 2 | 3 | 4 | 5 |
| --- | --- | --- | --- | --- | --- |
| ZN | 0.0226 | 0.0165 | 0.0127 | 0.0065 | 0.9417 |
| PD | 0.0259 | 0.0051 | 0.0164 | 0.9338 | 0.0189 |
| QL | 0.0058 | 0.0571 | 0.9184 | 0.0055 | 0.0131 |
| WM-1 | 0.6676 | 0.0922 | 0.1208 | 0.0293 | 0.0901 |
| WM-2 | 0.7269 | 0.2130 | 0.0075 | 0.0165 | 0.0362 |
| WM-3 | 0.2430 | 0.7109 | 0.0089 | 0.0113 | 0.0259 |

**Fig. S1** Principal coordinate analysis (PCoA) for 65 plant samples of *R. pudingense*


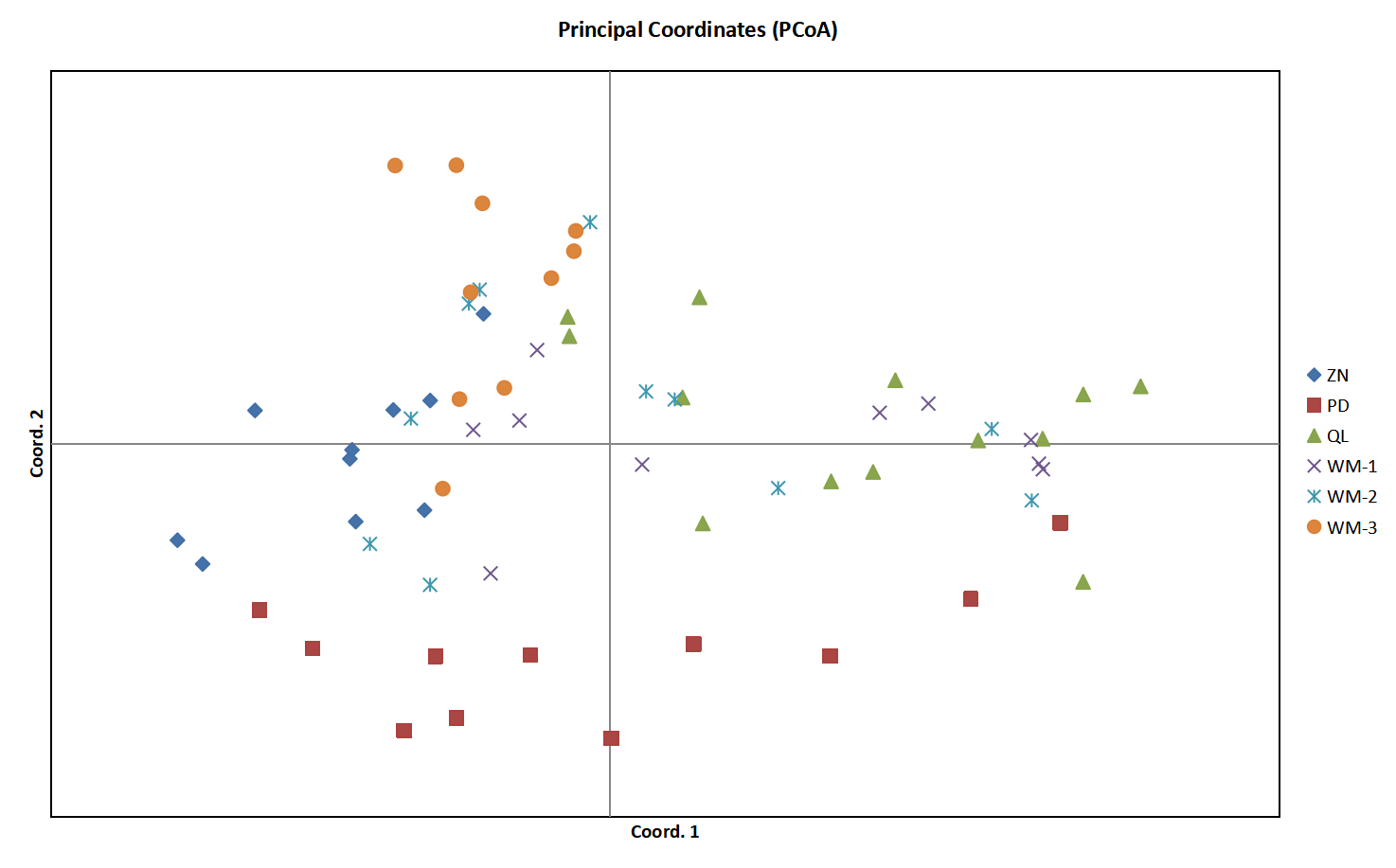

Supplement: Supplementary file 1 — Supplementary Material 1 [file 12870_2024_4759_MOESM1_ESM.docx]
